# Efficacy of Instagram in Promoting Psychoeducation in the Chinese-Speaking Population

**DOI:** 10.1089/heq.2019.0078

**Published:** 2020-04-02

**Authors:** Nikki H.T. Lam, Benjamin K.P. Woo

**Affiliations:** ^1^ College of Medicine, Northeast Ohio Medical University, Rootstown, Ohio, USA.; ^2^ Department of Psychiatry, Olive View-UCLA Medical Center, Sylmar, California, USA.

**Keywords:** Chinese, Instagram, first-episode psychosis, schizophrenia, YouTube

## Abstract

**Purpose:** To evaluate the efficacy of the use of Instagram in disseminating information regarding first-episode psychosis and schizophrenia.

**Methods:** Facebook and Instagram advertisements linked to an external YouTube video detailing first-time psychosis were initiated for 48 h. Metrics regarding the number of unique individuals reached and number of engagements were collected. Descriptive statistics were used to analyze the data.

**Results:** Facebook made 85 impressions (32.82%) and Instagram made 174 impressions (67.18%). Facebook had 24 engagements, whereas Instagram had 42.

**Conclusion:** Instagram is noninferior to Facebook in disseminating psychoeducational material to the Chinese-speaking population.

## Introduction

Schizophrenia is a debilitating mental health illness often characterized by a stepwise decline in social and cognitive function. Declines in function are triggered by psychotic episodes, and can typically be managed and prevented with antipsychotic medications.^1^ It is, therefore, important to recognize the symptoms and onset of psychosis to prevent further deterioration of a patient's quality of life.

Chinese populations face additional cultural and language barriers in the identification of a psychotic episode.^2^ Overall, 2.5% of the Chinese population has had a psychotic disorder during his or her lifetime.^3^ This suggests that a large number of cases of psychosis go untreated in this population. It is, therefore, imperative to make inroads in the delivery of culturally sensitive and effective mental health education to the Chinese population.

Previous studies have demonstrated the effectiveness of psychoeducation delivery through YouTube and Facebook in the Chinese population.^2,4,5^ However, little is known about the effect of information dissemination through Instagram. This study, therefore, aims to evaluate the usefulness and performance of an Instagram advertisement publicizing a YouTube video on first-episode psychosis knowledge with the Chinese-speaking populations.

## Methods

A video recording of a medical education talk show hosted by a board-certified psychiatrist and the radio station KMRB AM1430 in Los Angeles was uploaded to YouTube. This video provided psychoeducation in the Chinese language, and focused on the signs and symptoms of first-break psychosis.^2,6^ Institutional Review Board approval was not required as this was a descriptive study with minimal risk to the participants.

Facebook and Instagram advertisements written in the Chinese language were initiated, providing links to the YouTube video. The target population consisted of users of age 18–34 years, as this was the most likely presenting ages for first-episode psychosis. Each campaign lasted for 48 h.

A cross-sectional analysis was performed using analytics provided by both social media platforms. The reach (defined as the number of unique viewers), impression (defined as the total number of views), and engagements (defined as the number of clicks the ad generated) were recorded. Descriptive statistics were used to provide further analysis. The frequency of targeting new and unique users between the social media platforms was calculated by dividing the reach by the impressions. The frequency of generating an engagement per impression was also calculated.

## Results

All users viewed the advertisements from mobile devices. Overall, the advertisements together were viewed 259 times (impressions), and reached 252 unique viewers (reach). There were 85 Facebook impressions (32.82%) and 174 Instagram impressions (67.18%). Out of these, 79 unique subjects (31.35%) were Facebook users, whereas 173 unique subjects (68.65%) were Instagram users. Facebook resulted in 24 engagements, whereas Instagram resulted in 42. The frequency of targeting unique users on Facebook was 0.9294. On Instagram, the frequency was 0.9943. Finally, the frequency of engagement per impression (engagement rate) for Facebook was 0.2824, and for Instagram was 0.2414.

A total of $6.52 was spent on the advertisements, with $2.78 spent on Facebook and $3.74 spent on Instagram. The price for an engagement on Facebook was $0.11, and on Instagram was $0.09.

## Discussion

Previous studies have already established the efficacy of Facebook in providing exposure to psychoeducational materials.^2,4,5^ The results of this study suggest that Instagram is also just as effective in providing publicity. During the 48-h interval during which advertisements were run, Instagram delivered more impressions and reach overall, as well as more overall engagements. As such, the price per engagement on Instagram, despite initially spending more to host the advertisement, was lower. Instagram was also better at targeting unique users during its advertisement campaign. However, Facebook was marginally better at providing engagements per impression.

Strong stigma against mental health disorders exists across various Chinese culture, including Hong Kong Chinese and Singapore Chinese.^7,8^ It has been proven that informational campaigns are effective in combating societal discrimination.^9^ Our study suggests that the creative use of existing social media platforms may be an effective means of advertising existing informational material to difficult-to-reach populations. Future studies are required to more thoroughly characterize the population reached by our study, as well as the overall effect of the promotion of psychoeducation in the population.

A limitation to this study includes the inability to verify the actual population viewing the medium. The authors of this study assumed that viewers were Chinese speaking based on the content of the advertisement—non-Chinese readers would be more likely to ignore the advertisement. It is also important to note that our sample population was limited due to the fact that Facebook and Instagram are unusable in mainland China. However, this also suggests that our audience may not be limited to Chinese alone, but also other Chinese-speaking regions, including Hong Kong, Singapore, and the United States. In addition, the engagement rates for both social mediums are high enough to assume that the Facebook and Instagram algorithms selectively displayed the ad to users known to interact with content written in the Chinese language. Another limitation is the short campaign duration of 48 h. Also, it is unknown if users continued to view the video after engaging with the advertisement. Previous studies have suggested an average viewing time of around 4 min for psychoeducational YouTube videos.^10^ Future studies would benefit from a longer campaign duration with quick question surveys to further characterize sample demographics and to evaluate engagement time with advertised content.

## Conclusion

Indeed, as technology offers more cohesive and interconnected social mediums, better methods of psychoeducational outreach are being developed. Although the engagement rate was lower on Instagram, the data show that the overall number of engagements within the 48-h time frame was much greater than that of Facebook's. As such, Instagram may prove to be an equally effective, if not more, outreach medium than Facebook, particularly for the younger population.
